# Functional identity enhances aboveground productivity of a coastal saline meadow mediated by *Tamarix chinensis* in Laizhou Bay, China

**DOI:** 10.1038/s41598-020-62046-3

**Published:** 2020-04-02

**Authors:** Shijie Yi, Pan Wu, Xiqiang Peng, Fenghua Bai, Yanan Gao, Wenxin Zhang, Ning Du, Weihua Guo

**Affiliations:** 1 Institute of Ecology and Biodiversity, School of Life Science, Shandong University, Qingdao, 266237 People’s Republic of China; 2 Shandong Provincial Engineering and Technology Research Center for Vegetation Ecology, Qingdao, 266237 People’s Republic of China; 3 Shandong Academy of Forestry, Jinan, 250014 People’s Republic of China

**Keywords:** Biodiversity, Biodiversity, Biodiversity, Biodiversity, Community ecology

## Abstract

Research in recent decades has confirmed that biodiversity influences ecosystem productivity; however, the potential mechanisms regulating this process remain subject to controversy, due to variation across ecosystems. Here, the effects of biodiversity on ecosystem productivity were evaluated using three variables of biodiversity (taxonomic diversity, functional identity, and functional diversity) and surrounding environmental conditions in a coastal saline meadow located on the south coast of Laizhou Bay, China. At this site, the shrub and field layers were primarily dominated by *Tamarix chinensis* and natural mesic grasses, respectively. Our results showed that functional identity, which is quantified as the community weighted mean of trait values, had greater explanatory ability than taxonomic and functional diversity. Thus, ecosystem productivity was determined disproportionately by the specific traits of dominant species. *T. chinensis* coverage was a biotic environmental factor that indirectly affected ecosystem productivity by increasing the community weighted mean of plant maximum height, which simultaneously declined with species richness. The present study advances our understanding of the mechanisms driving variation in the productivity of temperate coastal saline meadows, providing evidence supporting the “mass ratio” hypothesis.

## Introduction

With the increasingly severe decline in biodiversity, it is crucial to evaluate the underlying consequences on ecosystem functioning caused by biodiversity loss^1^. Hence, the relationship between biodiversity and ecosystem functioning has been a hot and controversial topic in ecology in recent decades^2–5^, especially when considering ecosystem productivity across different vegetation types^6–9^.

Initial studies on biodiversity-productivity relationships mainly focused how taxonomic attributes (i.e. species richness as a traditional proxy of biodiversity) affect productivity^10–13^. One study that reviewed hundreds of articles showed that the effects of taxonomic diversity on productivity cannot be predicted, with the underlying mechanism being equally complex^14^. However, there is increasing evidence that functional traits represent the functional dissimilarity among species that coexist in a given community^15,16^, and that they are closely associated with niche difference processes. Thus, functional traits might have a stronger predictive power than taxonomic diversity on the biodiversity-productivity relationship^6,8,15,17^. Several recent studies have also elucidated a clear link between productivity and the physiological traits of dominant species^4,16^, highlighting the relevance of the trait-based approach to explain the variation in community productivity^18,19^.

Two conceptually different, but not mutually exclusive, mechanisms have emerged to explain how biodiversity affects ecosystem productivity: (1) selection and (2) complementarity effects. Selection effects influence biomass accumulation determined by the dominance of species^20^ with the highest yield or its functional traits in a community^17^. Specifically, ecosystem biomass is mainly determined by its functional identity, which is quantified as the community weighted mean (CWM) of trait values and is supported by the “mass ratio” hypothesis^6,21–23^. Some studies suggest that the “mass ratio” hypothesis is the foundation of the relationships between functional attributes and ecosystem productivity^6,24,25^. The different dimensions of diversity, such as taxonomic diversity and functional diversity, enhance productivity through the mechanism of complementary resource use in more diverse communities, which is described as complementarity effects^2,26^. The complementarity effects are supported by studies which have observed markedly positive relationships between diversity and productivity^27,28^.

The environment drives the relationship between biodiversity and ecosystem productivity^3,7,29,30^. In particular, primary productivity is influenced by soil fertility when sampling across highly variable environmental conditions^3,4^. The life-history strategies of species are influenced by the surrounding environment, leading to systematic changes of particular trait values along the environmental gradient^18,31,32^, thereby linking surrounding environment and community productivity^31^. This phenomenon could explain a significant amount of variation in productivity^2,3,21,33^.

This study aimed to determine how different dimensions of biodiversity influence productivity due to the impact of environmental factors. Specifically, we evaluated the effects of three different dimensions of biodiversity (taxonomic diversity, functional identity, and functional diversity), soil properties, and aboveground biomass of a coastal saline meadow located on the south coast of Laizhou Bay, China. We tested which biodiversity dimension best explains variation in the productivity of the saline meadow, and whether it reflected selection or complementarity effects. We also tested how environmental factors mediate biodiversity-productivity relationships, and the potential processes that influence the biomass dynamics of the coastal meadow in our study region.

## Results

### Predictors of biotic and environmental variables

*Tamarix chinensis* coverage was used as a biotic environmental factor that explained large amounts of variation in aboveground biomass of meadows (AGB), while none of the environmental factors were significant in the multiple linear regression analysis (Table 1). Species richness was better at explaining variation in AGB than species evenness for the taxonomic diversity variables (Table 1). For the functional attributes of community, we found that the CWM of plant maximum height (H_max_) and the functional dispersion (FDis) of leaf dry matter content (LDMC) were the most important predictors explaining AGB accumulation for the functional identity and functional diversity variables, respectively (Table 1). Therefore, based on multiple linear regression and model averaging, *T. chinensis* coverage, species richness, CWM of H_max_, and FDis of LDMC best predicted AGB in the coastal saline meadow.

**Table 1:** Results of the multiple linear regression and model averaging of environmental, taxonomic diversity, functional identity, functional diversity variables, and aboveground biomass. SW, sum of weight; SMC, soil moisture content; EC, electrical conductivity; TN, total nitrogen; TP, total phosphorus; CEC, cation exchange capacity; OC, organic carbon; AN, available nitrogen; EP, extractable phosphorus; AK, available kalium. CWM, community weighted mean; FDis, functional dispersion index; Hmax, maximum height; SLA, specific leaf area; LDMC, leaf dry matter content; LNC, leaf nitrogen concentration; LPC, leaf phosphorus concentration; SM, seed mass. The predictors selected are presented in bold. Variables with variance inflation values greater than three are not displayed.

Variable sets	Variables	SW	Estimate value	Standard error	95% confidence interval	*p*
Environmental	SMC	0.24	0.073	0.136	(−0.194, 0.341)	0.604
	EC	0.49	−0.182	0.127	(−0.430, 0.067)	0.152
	TN	0.30	0.095	0.163	(−0.225, 0.415)	0.573
	TP	0.28	−0.113	0.131	(−0.371, 0.144)	0.396
	CEC	0.27	−0.111	0.152	(−0.409, 0.186)	0.472
	OC	0.28	0.102	0.148	(−0.188, 0.392)	0.499
	AN	0.24	0.001	0.124	(−0.242, 0.244)	0.993
	EP	0.24	−0.004	0.130	(−0.259, 0.251)	0.977
	AK	0.30	−0.105	0.151	(−0.401, 0.192)	0.499
	***T. chinensis*** **coverage**	**0.68**	**0.261**	**0.138**	**(**−**0.009, 0.531)**	**0.058**
Taxonomic diversity	**Species richness**	**0.90**	−**0.286**	**0.114**	**(**−**0.509**, −**0.064)**	**0.012**
	Species evenness	0.71	−0.218	0.114	(−0.441, 0.004)	0.054
Functional identity	**CWM.H**_**max**_	**1.00**	**0.532**	**0.110**	**(0.316, 0.747)**	**<0.001**
	CWM.LDMC	0.32	−0.109	0.112	(−0.328, 0.111)	0.337
	CWM.N	0.25	0.018	0.124	(−0.226, 0.261)	0.896
	CWM.P	0.37	−0.143	0.127	(−0.392, 0.105)	0.262
	CWM.SM	0.25	−0.085	0.120	(−0.321, 0.151)	0.489
Functional diversity	FDis.H_max_	0.35	−0.121	0.126	(−0.367, 0.125)	0.340
	FDis.SLA	0.40	−0.089	0.151	(−0.385, 0.207)	0.568
	**FDis.LDMC**	**0.74**	**0.359**	**0.148**	**(0.069, 0.648)**	**0.015**
	FDis.N	0.69	−0.274	0.170	(−0.607, 0.058)	0.106
	FDis.P	0.38	−0.143	0.153	(−0.442, 0.156)	0.354
	FDis.SM	0.41	−0.117	0.148	(−0.406, 0.173)	0.438

### Correlation between predictors and AGB

**Figure 1:**
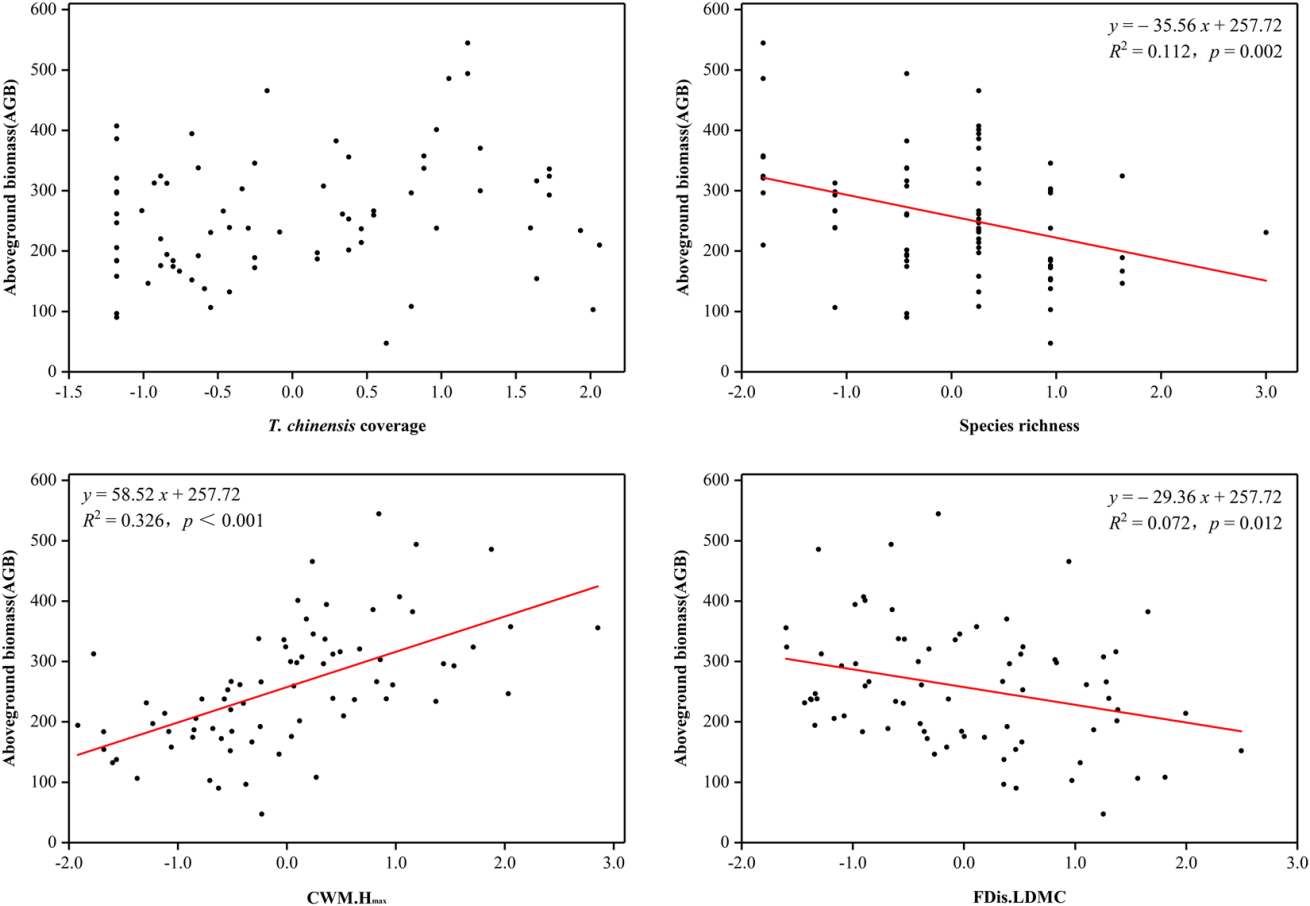
Relationship between aboveground biomass (AGB) and standardised predictors (*T. chinensis* coverage, CWM of maximum height, FDis of LDMC and species richness) in the coastal saline meadow located on the south coast of Laizhou Bay, China. *T. chinensis*, *Tamarix chinensis*; CWM, community weighted mean; FDis, functional dispersion index; LDMC, leaf dry matter content. Significant relationships are marked as solid lines (*p* < 0.05).

All biodiversity predictors were particularly sensitive to AGB compared to the selected environmental predictor, *T. chinensis* coverage (Fig. 1). Specifically, the CWM of H_max_ had a relatively and strongly positive correlation with AGB (*R*^2^ = 0.326, *p* < 0.001). In comparison, species richness (*R*^2^ = 0.112, *p* = 0.002) and the FDis of LDMC (*R*^2^ = 0.072, *p* = 0.012) had significantly negative effects on AGB. However, no significant effect was detected between *T. chinensis* coverage and AGB.

### Structural equation model

**Figure 2:**
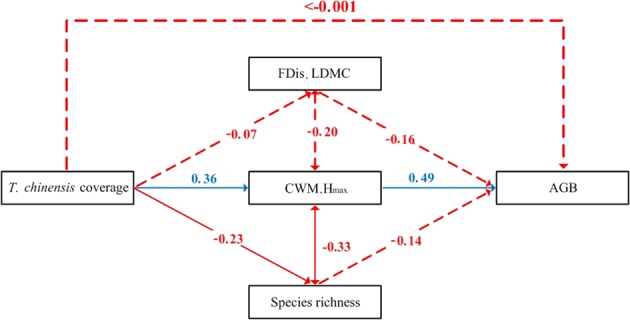
Structural equation model testing the relationship between biodiversity predictors and aboveground biomass (AGB) on the change of *T. chinensis* coverage. For abbreviations of predictors, see Fig. 1. Significant impacts are marked as solid lines, whereas non-significant impacts are marked as dotted lines. Positive impacts are marked in blue, whereas negative impacts are marked in red. Covariance relationships are marked as lines with a two-way arrow.

The model that best fitted our observation data included AGB, species richness, CWM of H_max_, FDis of LDMC, and *T. chinensis* coverage (χ^2^ = 0.800, *p* = 0.371, RMSEA < 0.001, GFI = 0.995). AGB was positively and strongly correlated with the CWM of H_max_ (Fig. 2, λ = 0.49), while the FDis of LDMC and species richness had no significant direct effect on AGB. *T. chinensis* coverage was not affected by AGB directly, but it largely promoted the CWM of H_max_ (Fig. 2, λ = 0.36) and contributed to a clear decline in species richness (Fig. 2, λ = −0.23).

## Discussion

This study identified the key predictors of biodiversity that drive dynamic changes to productivity, elucidating how biodiversity influences productivity in saline meadow. Specifically, we showed that selection effects strongly influenced ecosystem productivity, while complementarity effects did not. In particular, the maximum height of dominant species significantly enhanced productivity mediated by *T. chinensis* coverage.

The environment was hypothesised to be primarily important for productivity in the resource-limited habitat, and should, therefore, provide better predictive power than measurements of biodiversity^3^. Unexpectedly, none of the univariate edaphic variables captured the variation in AGB. However, *T. chinensis* coverage served as an appropriate proxy for environmental variables in SEM, indicating that *T. chinensis* influences meadow species, but with no clear direct effect. Previous work reported that *T. chinensis* exhibits the facilitation effect on coexisting herbaceous species in the same region^34,35^. *T. chinensis* transfers nutrients from the deep soil layer to the surface, producing the “fertile island” to enhance the available nutrients for meadow species^34^. Species richness represented an important and logical approach for estimating the realised niche differentiation. Theoretically, this parameter, is related to the extent of niche differentiation in biomes^12^. Furthermore, species richness showed better independence from other biodiversity indexes than evenness (see Appendix S1). This parameter might independently capture variation in AGB well. For the functional identity variables, the CWM of H_max_ was vital for AGB, rather than leaf and seed traits. Maximum plant height, was an indicator for competition for available resources (e.g. nutrients, light)^36^. It was also important for the biomass production of plants^37^. The CWM of H_max_ was correlated with the plant growth of the most abundant species, and represented one of the main drivers of biomass in grasslands and forests^25,38^. For the measurements of functional diversity, the FDis of LDMC was associated with AGB. LDMC was an independent strategy axis related to plant tolerance for stressful environment, indicating that conservative strategy and environment tolerance are important for biomass production in saline meadows^36^.

As the environmental predictor, *T. chinensis* coverage did not improve AGB in the linear regression. However, *T. chinensis* coverage improved AGB through the functional identity variables, even though there was no direct correlation between *T. chinensis* coverage and AGB in SEM. In our study, we identified some soil fertility factors, including soil organic carbon, total nitrogen, available kalium, and cation exchange capacity. These factors were notably and positively related to *T. chinensis* coverage based on the Pearson correlation test (*r* = 0.4–0.7, see Appendix 1), indicating soil fertility increased with *T. chinensis* coverage, and confirming the existence of the facilitation effect. Previous studies also demonstrated that net facilitation effect was more likely to occur in stressful environments^39^. Specifically, the formation of fertile islands is an important process driving the positive interactions between shrubs and grass^39^.

For taxonomic diversity, our results indicated that species richness was negatively correlated to AGB in the linear regression, and lost significance when accounting for the selected environmental predictor, *T. chinensis*. Negative and non-significant correlations between species richness and AGB have been observed in previous studies^4,5,40,41^, with the opposite being found for complementarity effects. This negative association has been documented in fertilisation experiments^42^ and in communities with high productivity^5^. In our study, the distribution of resources was inequitable among species within meadow communities, when soil fertility conditions shifted from poor to benign with increasing *T. chinensis* coverage. Consequently, certain dominant species with a greater fitness advantage obtained more resources disproportionately in the saline meadow. This phenomenon might cause the competitive exclusion of subordinate species, leading to a decline in species richness, which is consistent with previous studies^5,40,43^.

The linear regression showed that the CWM of H_max_ explained the greatest variation in AGB, with this significant correlation also being obtained in the SEM that included the selected environmental predictor, *T. chinensis*. This result was consistent with many previous studies in diverse ecosystems^8,15,41^, indicating that selection effects drive ecosystem productivity. Our results suggest that productivity was primarily determined by the maximum height of the dominant plant species in the coastal saline meadow. Therefore, the functional identity performed better and was more sensitive in promoting AGB accumulation than other biodiversity dimensions, supporting the “mass ratio” hypothesis^2,21^. Additionally, *T. chinensis* coverage also improved the CWM of H_max_ in the SEM. In general, higher soil fertility was positively associated with more efficient photosynthesis and a higher relative growth rate of grasses. This process, in turn, promoted primary production, leading to dominant species having disproportionate benefits^21^. The dominant species obtained more nutrient from the soil, which led to increased biomass and maximum height. This phenomenon was consistent with that of studies conducted in the Inner Mongolia grasslands^15^ and the Tibetan alpine meadows^42^ of China, indicating that the maximum height of the dominant species markedly improved with increasing soil fertility. Simultaneously, increasingly limited light availability to subordinate grasses, as a result of higher height of dominant grasses, intensified competition for light within the meadow community. The CWM of H_max_ was a good predictor for intensive competition for light when nutrient availability increased^36,44^. This result was supported by previous work showing that increased CWM of H_max_ promotes community productivity by enhancing light capture^45^. In addition, we found that the functional identity variable explained biomass dynamics more than taxonomic diversity. This finding was consistent with the results of studies in temperate grasslands^6^ and forest ecosystems^46^, which showed that functional identity is a more efficient predictor of ecosystem productivity than taxonomic diversity.

Of the functional diversity variables considered, the FDis of the LDMC exhibited a more convergent pattern to promote AGB in the linear regression but had no significant correlation with AGB in the SEM. Thus, increased variation in LDMC might have a negative influence on productivity, supported by previous work^47^. Species survival tended to be a conservative life-history strategy, showing a pattern of functional convergence in response to strong environmental pressures via higher LDMC^48^. These results were supported by the findings of studies in temperate forests, which showed that trees promote carbon storage by improving stem specific density^4,9^. This phenomenon arises because LDMC was closely related to stem specific density, which is considered to be an analogical index to stem specific density in herbaceous species^49^. However, many studies have stated that functional diversity enhances productivity through optimal resource use^1,50^. In particular, because barren environments have a weak competition intensity, complementarity effects are more likely to drive productivity^7^. Yet, our results showed the opposite effect, in which functional dispersion was negatively correlated with AGB. This phenomenon is probably explained by the presence of highly productivity species that had similar specific trait values and low-levels of community functional dispersion^51^ in the stressful environment. Thus, complementarity effects had a negligible role in driving variation in AGB in our study.

Our results show that *T. chinensis* clearly promotes the maximum height of dominant species and decreases species richness, which, in turn increases the productivity of saline meadows indirectly in the temperate coastal zone. Our study demonstrates the important role of functional identity and selection effects on the relationship between biodiversity and ecosystem productivity, supporting the “mass ratio” hypothesis. Our study indicates that conservation measures should concentrate on protecting woody species that facilitate grasses to optimise the production of saline meadows in temperate coastal zones.

## Methods

### Study area

The natural coastal saline meadow examined in this study is located in a marine reserve (37°03′–37°07′N; 119°20′–119°23′E) in the south part of Laizhou Bay, China. The mean annual temperature of the study area is 12 ^o^C and the mean annual precipitation is 630 mm. In this region, we can find the youngest coastal wetland ecosystems in China, with dramatic changes in environment and landscape from shoreline to inland area^52,53^. The saline meadow is the most important vegetation type in this region which contributes towards stabilising sandy habitat and preventing seawater encroachment. *T. chinensis* is the dominant species of shrub and the only arboroid species within the study area. This species, has been previously confirmed to facilitate the growth of herbaceous plants by improving the availability of nutrient resources in microhabitats^34,35^. For the herb layer, *Artemisia capillaris*, *Artemisia scoparia*, *Setaria viridis* and *Conyza Canadensis* were the dominant species in the study area. Moreover, our study area encompassed a broad environmental gradient, extending from the shoreline to inland area. Consequently, the productivity and species composition of biomes noticeably changed. Therefore, it is necessary to examine how biodiversity influences productivity in coastal wetland ecosystem.

### Establishment of plots

**Figure 3:**
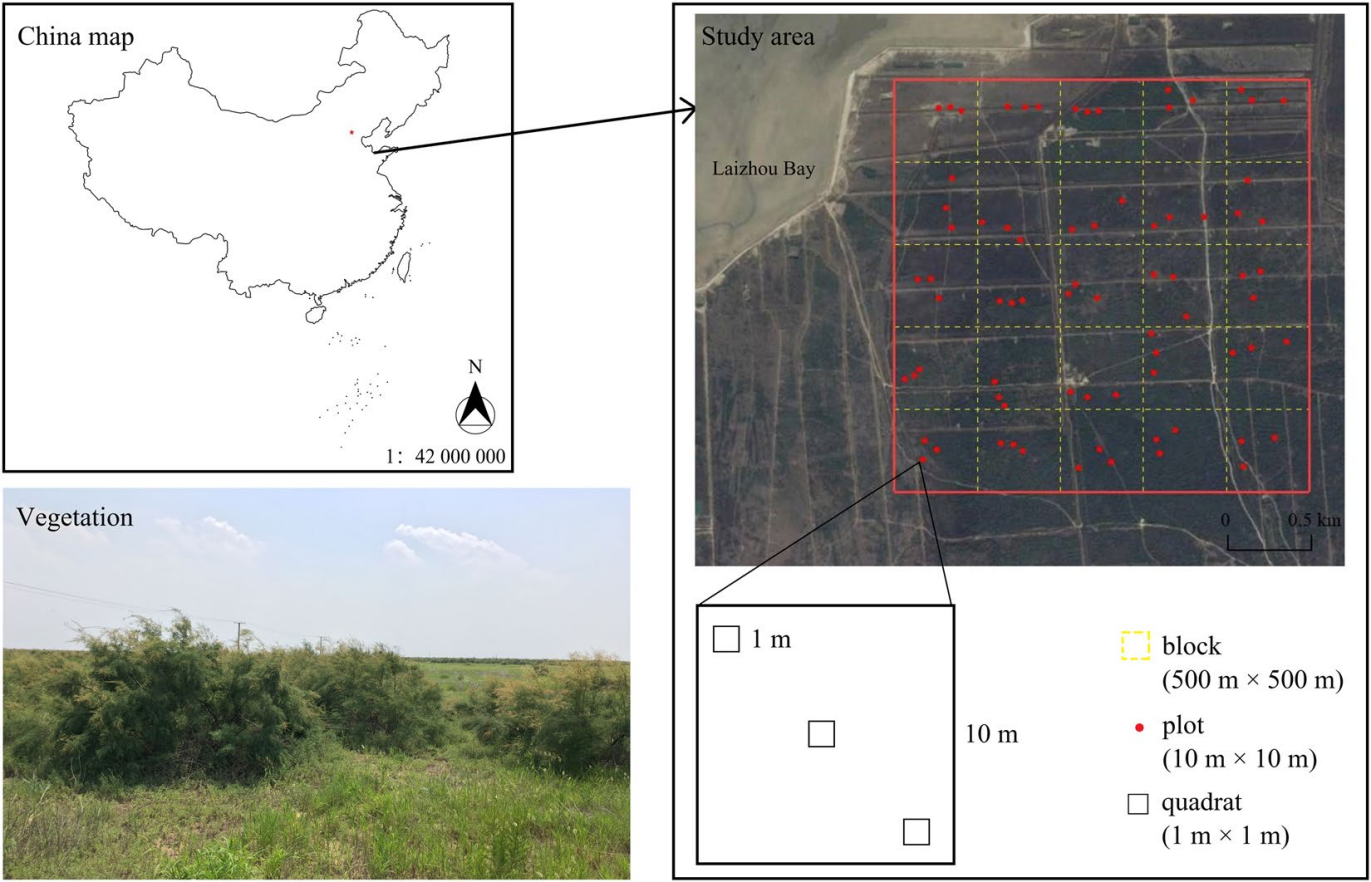
Location of study area and sampling plots on the south coast of Laizhou Bay, China. The study area is indicated as a red quadrangle, the grid blocks are indicated as yellow dotted quadrangles, and the sampling plots are indicated as red points. Three plots (10 m × 10 m) are established within each block and three quadrats are nested (1 m × 1 m) in each plot. The satellite imagery is obtained from Google Earth (Version 7.3.0), 2020 CNES/Airbus (https://www.google.com/earth/).

We separated a 2.5 km × 2.5 km area into twenty-five 500 × 500 m grid blocks in the core of the reserve (Fig. 3), that has never been cultivated, but it has been disturbed artificially by the construction of canals and roads. For each block, three plots (10 m × 10 m) were established to characterize the *T. chinensis* in terms of coverage, average height and the number of branches that was at least 50 m from the closest artificial facilities (canal or roads), resulting in 75 plots in total. Within each plot, three quadrats (1 m × 1 m) were established at the centre and the opposite two corners of a plot that were not shaded by the *T. chinensis* canopy.

In each quadrat, all herbaceous plants were taxonomically identified and the coverage per species was recorded. Subsequently, we harvested all aboveground biomass per quadrat to assess the primary productivity of the saline meadow community at the end of the growing season (August to September 2017). Because the biomass has reached the annual peak during sampling, the aboveground biomass is close to the net primary productivity of saline meadow^54^. We calculated the aboveground biomass of meadows (AGB) at the plot level as an average mass following drying at 80 ^o^C for 48 h. The AGB ranged from 47.61 g/m^2^ to 544.89 g/m^2^, with an average of 257.72 g/m^2^. Because we lost one AGB dataset, we implemented the subsequent analyses on the data from the remaining 74 plots.

### Trait measurements

We chose six functional traits to assess different functional strategies for each species that was identified. Specifically, maximum plant height was associated with a strategy axis of competition; leaf traits (specific leaf area, leaf dry matter content, leaf nitrogen and phosphorus concentration) represented the leaf economics spectrum, and were related to an acquisitive or conservative resource use strategy; and seed mass was correlated with dispersal capability^36,55^. With the exception of seed mass, each functional trait was measured for at least 10 individual replicates per species sampled in each plot to incorporate intraspecific variation into trait measures. This is because intraspecific trait variation influences community composition and, hence, ecosystem productivity^32^. We collected and measured functional traits in the plots by following standardised protocols^56^. For overall plant traits, we used maximum plant height (H_max_), which was determined as the shortest distance between the upper boundary of main photosynthetic tissues and the ground. For the leaf morphologic traits, we chose mature and healthy leaves exposed to sunshine with petioles and rachis to calculate specific leaf area (SLA) and leaf dry matter content (LDMC). SLA was calculated as the rehydrated leaf area divided by its oven-dried mass. LDMC was calculated as the oven-dry mass divided by its fresh mass. To determine leaf chemical traits, leaves without any petiole or rachis were collected, oven-dried, and ground before measuring leaf nitrogen concentration (LNC) and leaf phosphorus concentration (LPC), respectively. Seed mass (SM) was based on the oven-dried mass of 1000 seeds obtained from both the study area and the Germplasm Bank of Wild Species in Southwest China (http://www.genobank.org). This parameter represented a species-level trait value.

### Environmental variables

For each plot, 12 biotic and abiotic environmental variables were collected and quantified in our study. Three biotic environmental variables influencing the growth of meadows, including *T. chinensis* coverage, average height, the number of branches, were obtained from the field site. We used *T. chinensis* coverage as a proxy for biotic variables because it was closely associated with AGB and was strongly correlated with other biotic variables. Among the abiotic variables, we measured nine soil properties, including soil moisture content (SMC, g/g), electrical conductivity (EC, μS/cm), total nitrogen (TN, mg/kg), total phosphorus (TP, mg/kg), cation exchange capacity (CEC, cmol/kg), organic carbon (OC, mg/kg), available nitrogen (AN, mg/kg), extractable phosphorus (EP, mg/kg), and available kalium (AK, mg/kg). Three replicates were obtained at each quadrat and were combined to form a single sample per plot. These variables were used to assess the diversity of coastal soil properties, including water utilisation, salinity gradient, and soil fertility. These parameters are essential for the ecosystem processes of coastal saline meadows. Every environmental variable was standardised by subtracting its mean and then divided it by the standard deviation for the subsequent analysis.

### Quantification of biodiversity variables

To predict variation in AGB, we focused on biodiversity levels, which were grouped into two categories: functional attributes and taxonomic attributes. Functional attributes were further categorised into functional identity and functional diversity.

Taxonomic variables were computed as two measures: species richness and species evenness. Both measures represented taxonomic diversity to evaluate the effects of biodiversity on ecosystem productivity^30,46,57^. Functional identity was quantified as the community weighted mean (CWM) trait value of a single continuous trait separately. The calculation was based on the species relative abundance and trait values,

$${\rm{CWM}}=\mathop{\sum }\limits_{i=1}^{n}{p}_{i}\times trai{t}_{i}$$

where *p*_*i*_ is the relative abundance of species *i*; *trait*_*i*_ is the value of a specific trait of species *i*; and *n* is the number of species^58^.

This parameter was an aggregated value of given species and its relative abundance in a plot^20^. The CWM trait value was widely used in biodiversity-ecosystem functioning studies, potentially representing the most sensitive indicator of biodiversity composition on ecosystem functioning, because ecosystem functioning is disproportionately determined by the more abundant species that is correlated to the mechanism of selection effects^3,6,9,24^. Since all trait data are continuous variables and the values are greater than 0, the logarithmic transformation is used to make the trait data follow the normal distribution prior to statistical analysis. We used trait values at the plot scale and considered plot-level intraspecific trait variability in calculation. For functional diversity variables, we calculated functional dispersion (FDis) indices based on a log-transformed monoculture trait value to assess community functional diversity. This variable is rarely correlated with species richness; thus, it was used to ensure that functional diversity variables were independent of taxonomic diversity variables^59^. Indeed, we found that functional dispersion had low correlations with species richness (Appendix 1). Moreover, single-trait functional dispersion indices have been shown to capture important information on trait variation across the environmental gradient^60^, as well as in ecosystem processes^61,62^. In addition, to avoid bias in the results of the selected indices, we calculated Rao’s Quadratic entropy index, which was calculated as the abundance-weighted functional distance of two random individuals in a community^59,63^. Because Rao’s Q index was highly correlated with functional dispersion indices, we did not describe these results further. All functional diversity indices were calculated using the R package ‘FD’^59^.

### Statistical analyses

We applied structural equation modelling (SEM) to test the direct and indirect impacts of environmental factors and biodiversity variables on ecosystem productivity. Our conceptual model was constructed using existing knowledge of biodiversity-ecosystem functioning, validated by the observational data of previous studies^3,5,64^. In our study, we tested three alternative pathways from biodiversity to AGB, which cause variation in AGB accumulation in the coastal saline meadow. As sample size was limited, the SEM based on our observational data required simplification^5,64^. Specifically, we only selected the best individual predictor per set of variables (environment, functional identity, functional diversity, and taxonomic diversity) that was expected to have the greatest influence on variation in AGB.

Multiple linear regression and multi-model inference analyses were conducted to evaluate the relative strength of different individual factors on AGB simultaneously. For these analyses, we used the original AGB data that were normally distributed as dependent variables, and we standardised all biodiversity factors as independent variables. We excluded individual factors for which variance inflation factors scores were greater than three during the calculation to avoid notable and substantial multi-collinearity in the models^65^. The corrected Akaike Information Criterion (AIC_C_) was used to identify model performance^66^; that is, the best model had the lowest AIC_C_ value. In some cases, we obtained multiple candidate models that had differences of less than two units for the AIC_C_ with the lowest AIC_C_. Therefore, we conducted a full model averaging procedure to identify the best predictor for AGB per set of variables^66^. We calculated the full standardised effect size to evaluate which individual factor was the most sensitive predictor^67,68^. Factors with a 95% confidence interval of standardised effect size that excluded zero and had a sum of weight (SW) value greater than 0.9 were determined as the best predictors on variation of AGB^69^. If none of the factors met our criteria, we selected the factor with the largest SW value as the best predictor. In addition, we also tested exponential, lognormal, and unimodal correlations between different variables and AGB; however, we did not find any other function shape for the correlations. Multiple linear regression and multi-model inference analyses were conducted using the R package ‘MuMIn’^70^.

We examined the relationships between selected predictors and AGB using linear regression to evaluate biodiversity-productivity relationships. We then imported observational data into our conceptual SEM to assess the impacts of all predictors. The initial SEMs were conducted using the R package ‘lavaan’^71^, and included all predictors selected from the set of variables and AGB. We aggregated the existing covariant relationship between different biodiversity predictors into SEMs. We assessed the utility of SEMs using a Chi-square test, root mean square error of approximation (RMSEA), and goodness-of-fit index (GFI). The final SEM was adopted with a good fit (*p* > 0.05), a RMSEA < 0.05, and a GFI > 0.95^72^.

## Supplementary information

Supplementary information.

## Data Availability

The datasets analysed during the current study are available from the corresponding authors on request.
