# Supplementary material for: Functional identity enhances aboveground productivity of a coastal saline meadow mediated by Tamarix chinensis in Laizhou Bay, China
Source: Sci Rep. 2020 Apr 2;10:5826. doi: 10.1038/s41598-020-62046-3 (PMC7118169; doi:10.1038/s41598-020-62046-3)
Supplement: Supplementary file 1 — Supplementary information. [file 41598_2020_62046_MOESM1_ESM.docx]

Supporting information to the paper

**Functional identity enhances aboveground productivity of a coastal saline meadow mediated by *Tamarix chinensis*** **in Laizhou Bay, China**

Shijie Yi^1, 2^, Pan Wu^1, 2^, Xiqiang Peng^1, 2^, Fenghua Bai^1, 2^, Yanan Gao^1, 2^, Wenxin Zhang^3^, Ning Du^1, 2, *^, Weihua Guo^1, 2, *^

^1^ *Institute of Ecology and Biodiversity, School of Life Science, Shandong University, Qingdao, 266237, People’s Republic of China.*

^2^ *Shandong Provincial Engineering and Technology Research Center for Vegetation Ecology, Qingdao,* *266237, People’s Republic of China.*

^3^*Shandong Academy of Forestry, Jinan, 250014, People’s Republic of China.*

** is the corresponding author.*

Email: whguo_sdu@163.com

**Appendix S1**

**Table 1.** Pearson correlation coefficients between all standardised environmental variables. Significant impacts are marked in bold.

|  | Soil moisture content | Electrical conductivity | Total nitrogen | Total phosphorus | Cation exchange capacity | Organic carbon | Available nitrogen | Extractable phosphorus | Available kalium | *T. chinensis* coverage | *T. chinensis* average height | *T. chinensis* branch numbers |
| --- | --- | --- | --- | --- | --- | --- | --- | --- | --- | --- | --- | --- |
| Soil moisture content | 1.000 | 0.058 | -0.051 | -0.082 | **0.306** | -0.133 | 0.072 | 0.271 | **-0.081** | -0.117 | 0.003 | **-0.286** |
| Electrical conductivity | 0.058 | 1.000 | 0.219 | 0.089 | 0.063 | 0.207 | -0.055 | 0.311 | **0.018** | 0.161 | 0.184 | 0.183 |
| Total nitrogen | -0.051 | 0.219 | 1.000 | 0.181 | **0.297** | **0.533** | -0.019 | 0.432 | **0.073** | **0.402** | **0.333** | **0.403** |
| Total phosphorus | -0.082 | 0.089 | 0.181 | 1.000 | -0.228 | 0.077 | -0.041 | 0.094 | 0.072 | **0.274** | 0.083 | **0.263** |
| Cation exchange capacity | **0.306** | 0.063 | **0.297** | -0.228 | 1.000 | **0.233** | -0.060 | 0.385 | **0.240** | 0.011 | 0.072 | -0.114 |
| Organic carbon | -0.133 | 0.207 | **0.533** | 0.077 | **0.233** | 1.000 | -0.024 | **0.317** | **0.200** | 0.145 | 0.187 | 0.213 |
| Available nitrogen | 0.072 | -0.055 | -0.019 | -0.041 | -0.060 | -0.024 | 1.000 | -0.085 | 0.156 | -0.152 | -0.115 | -0.169 |
| Extractable phosphorus | 0.271 | 0.311 | 0.432 | 0.094 | **0.385** | **0.317** | -0.085 | 1.000 | 0.026 | 0.265 | 0.264 | 0.260 |
| Available kalium | **-0.081** | **0.018** | **0.073** | 0.072 | **0.240** | **0.200** | 0.156 | 0.026 | 1.000 | **-0.009** | **-0.043** | **0.009** |
| *T. chinensis* coverage | -0.117 | 0.161 | **0.402** | **0.274** | 0.011 | 0.145 | -0.152 | 0.265 | **-0.009** | 1.000 | **0.567** | **0.893** |
| *T. chinensis* average height | 0.003 | 0.184 | **0.333** | 0.083 | 0.072 | 0.187 | -0.115 | 0.264 | **-0.043** | **0.567** | 1.000 | **0.455** |
| *T. chinensis* branch numbers | **-0.286** | 0.183 | **0.403** | **0.263** | -0.114 | 0.213 | -0.169 | 0.260 | **0.009** | **0.893** | **0.455** | 1.000 |
